# Dual biologics for severe asthma and atopic dermatitis: Synopsis of two cases and literature review

**DOI:** 10.1002/rcr2.1266

**Published:** 2023-12-06

**Authors:** Takeshi Matsumoto, Yumiko Sakurai, Noriyuki Tashima, Tomoya Matoba, Akiko Kaneko, Takahiro Fujiki, Yusuke Kusakabe, Emi Nakayama, Ayaka Tanaka, Mayuko Tashima, Naoki Yamamoto, Kensaku Aihara

**Affiliations:** ^1^ Department of Respiratory Medicine Saiseikai‐Noe Hospital Osaka Japan; ^2^ Department of Dermatology Saiseikai‐Noe Hospital Osaka Japan

**Keywords:** asthma, biologic, combination, dupilumab, tezepelumab

## Abstract

The efficacy and safety of the combination of biologic therapies remain unclear with an ineffective and insufficient single biologic for managing asthma. Herein, we report two cases using dual biologics for severe asthma and atopic dermatitis. A 52‐year‐old male patient who received dupilumab and mepolizumab, benralizumab, or tezepelumab, followed by bronchial thermoplasty, and a 41‐year‐old male patient who received dupilumab and omalizumab, both experienced improved asthma and atopic dermatitis. To date, 38 cases are using dual biologics for severe asthma. The success rate was 84%, with no major adverse effects. We report the first case of severe asthma receiving dual biologics with tezepelumab and furthermore bronchial thermoplasty, and comprehensive literature review on dual biologics. Dual biologics may be an effective treatment method for severe asthma, requiring further investigation.

## Graphical abstract

Herein, we report two cases using dual biologics for severe asthma and atopic dermatitis. A 52‐year‐old male patient who received dupilumab and mepolizumab, benralizumab, or tezepelumab, followed by bronchial thermoplasty, and a 41‐year‐old male patient who received dupilumab and omalizumab, both experienced improved asthma and atopic dermatitis. Dual biologics may be an effective treatment method for severe asthma, requiring further investigation.

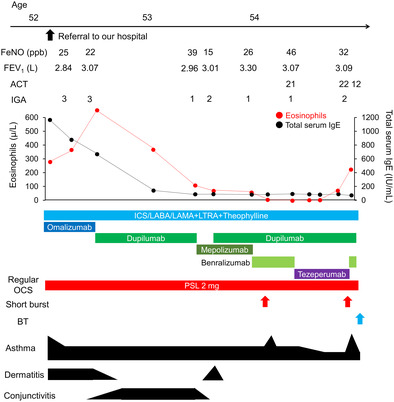

## INTRODUCTION

Biologics are a pivotal therapeutic approach for severe asthma, offering targeted interventions against several cytokines such as immunoglobulin E (IgE) (omalizumab), interleukin (IL)‐5 (mepolizumab, benralizumab, and reslizumab), IL‐4/IL‐13 (dupilumab), and thymic stromal lymphopoietin (TSLP) (tezepelumab).^1^, ^2^, ^3^, ^4^, ^5^ These biologics are widely used for treating not only severe asthma but also other allergic disorders, including chronic spontaneous urticaria, atopic dermatitis, and chronic rhinosinusitis.^6^, ^7^ Switching to an alternative biologic is recommended rather than resorting to combination therapy when a single biologic is ineffective or insufficient for managing asthma.^8^ However, modifying only one pathway for asthma pathophysiology might be insufficient, and a clinical situation sometimes works well with a combination of biologic therapies.

Several case reports and two case series detailed the use of dual biologics that were approved for severe asthma while clinical trials in this area remain unavailable to date.^9^, ^10^, ^11^, ^12^, ^13^, ^14^, ^15^, ^16^ Notably, one case series presented a favourable response in 11 out of 15 (73%) cases that received dual biologic therapy.^16^

Herein, we report two cases receiving dual biologics. Dupilumab is a good candidate for patients with severe asthma and atopic dermatitis; however, when dupilumab fails to effectively manage asthma and an alternative biologic lacks strength for atopic dermatitis, the potential of dual biologics, inclusive of dupilumab, becomes an enticing strategy. We also provide a comprehensive literature review on the combination of biologic therapies for severe asthma, thereby augmenting the existing evidence surrounding dual biologic interventions.

## CASE REPORT

### Case 1

**FIGURE 1:**
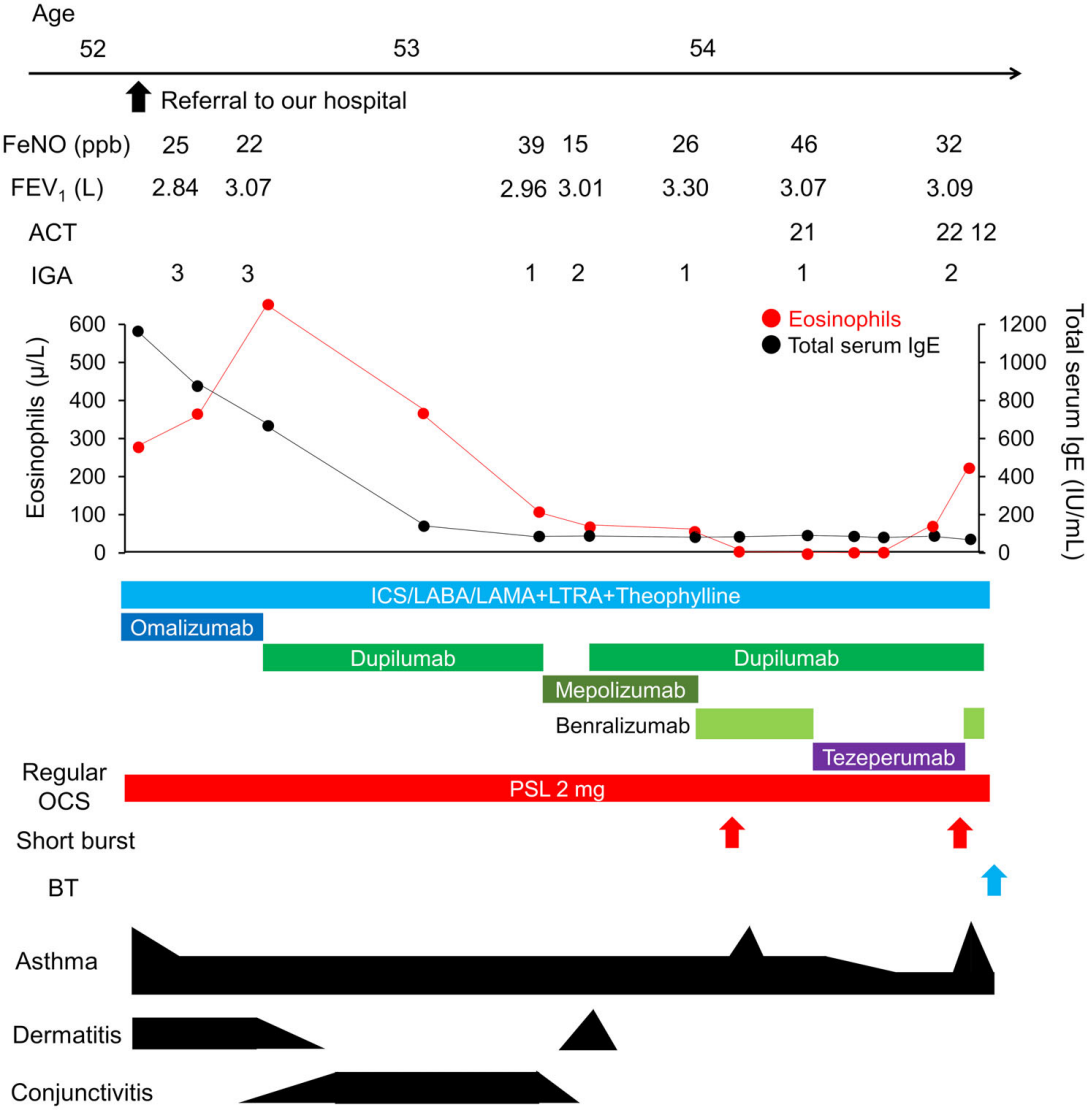
Clinical course in case 1. Black figure size represents the degree of symptoms of each situation. Red arrows represent the occurrence of short burst, while blue arrow represents the performance of BT. FeNO, fractional exhaled nitric oxide; FEV_1_, forced expiratory volume in 1 s. ACT, asthma control test; BT, bronchial thermoplasty; ICS, inhaled corticosteroid; IGA, investigator global assessment; IgE, immunoglobulin E; LABA, long‐acting beta2‐agonist; LAMA, long‐acting muscarinic antagonist; LTRA, leukotriene receptor antagonist; OCS, oral corticosteroid; PSL, prednisolone.

A 52‐year‐old male patient was referred to our hospital for severe asthma treatment. He had a history of childhood asthma, chronic spontaneous urticaria, and atopic dermatitis. He had never smoked and had no drug or food allergies. The following laboratory tests were evaluated: white blood cell count of 5700/mm^3^ with 4.9% eosinophils, total serum IgE of 1176 IU/mL, and specific IgE levels for house dust of 9.73 UA/mL. Omalizumab administration was initiated, which resolved asthma. He had been prescribed oral corticosteroids; however, tapering had been unsuccessful. Four months later, omalizumab was replaced with dupilumab, expecting additive improvement in atopic dermatitis. Unfortunately, this change triggered the onset and progression of conjunctivitis. Therefore, dupilumab was replaced with mepolizumab. This adjustment worsened dermatitis. Consequently, dupilumab was reintroduced with continuing mepolizumab. This combination effectively managed atopic dermatitis without deteriorating conjunctivitis. Nonetheless, asthma management remained suboptimal; therefore, mepolizumab was replaced with benralizumab. Regrettably, this alteration failed to ameliorate asthma. Therefore, benralizumab was replaced with tezepelumab, resulting in a 5‐month period of partially improved asthma management. However, a gradual elevation in eosinophil count and a loss of asthma management occurred. Consequently, tezepelumab was again replaced with benralizumab, and the patient underwent bronchial thermoplasty. At present, he continues benralizumab and dupilumab treatment (Figure 1). The use of dual biologics demonstrated no adverse effect associations.

### Case 2

**FIGURE 2:**
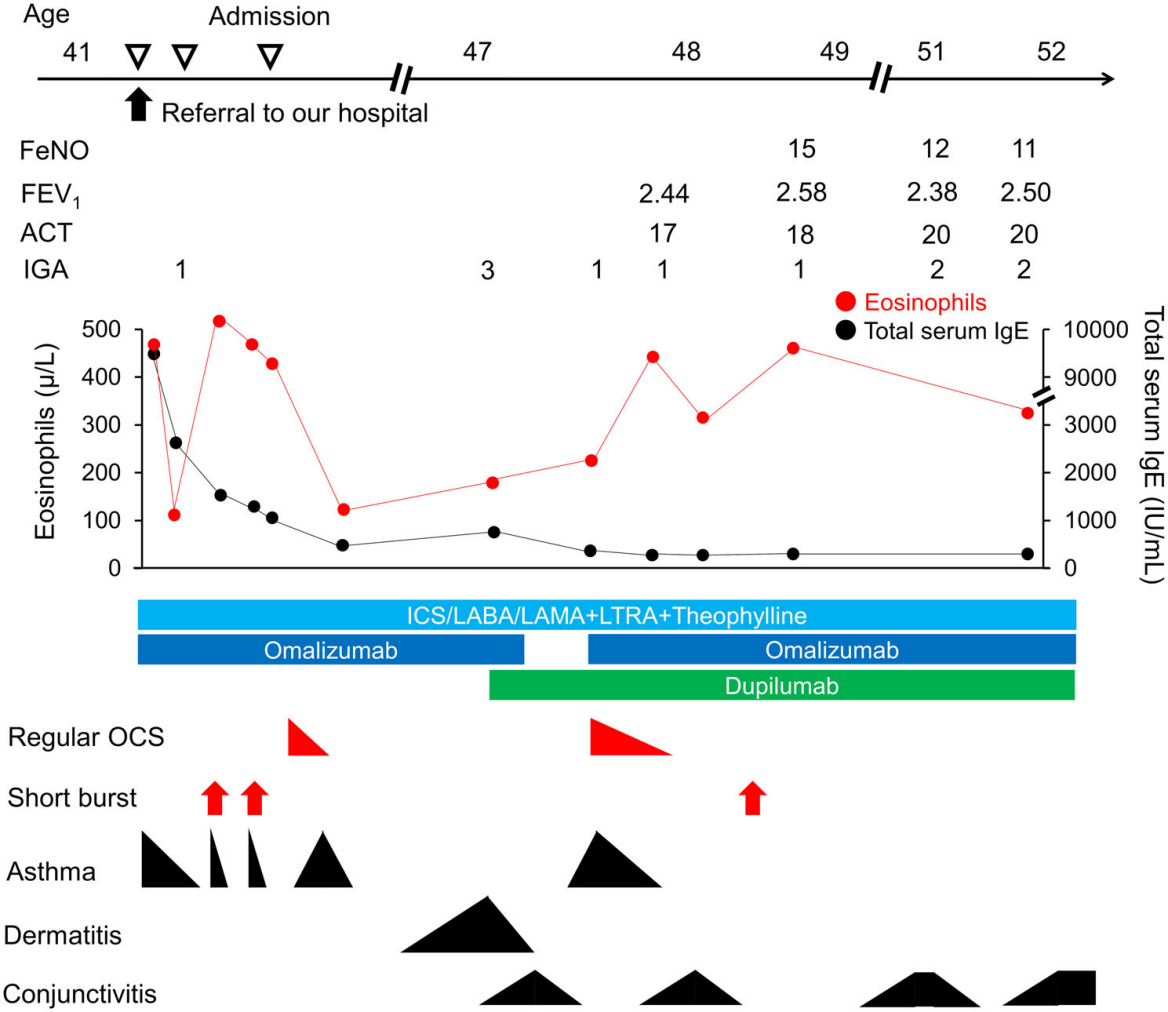
Clinical course in case 2. Black figure size represents the degree of symptoms of each situation. Red arrows represent the occurrence of short burst. FeNO, fractional exhaled nitric oxide; FEV_1_, forced expiratory volume in 1 s. ACT, asthma control test; ICS, inhaled corticosteroid; IGA, investigator global assessment; IgE, immunoglobulin E; LABA, long‐acting beta2‐agonist; LAMA, long‐acting muscarinic antagonist; LTRA, leukotriene receptor antagonist; OCS, oral corticosteroid.

A 41‐year‐old male patient was referred to our hospital for severe asthma treatment. He had a history of childhood asthma and atopic dermatitis. He had a history of 2 pack‐year smoke and had no drug or food allergies. The following laboratory tests were evaluated: white blood cell count of 11,200/mm^3^ with 4.2% eosinophils, total serum IgE of 9290 IU/mL, and specific IgE levels for house dust of 11.40 UA/mL. Omalizumab was initiated, which subsequently resolved asthma. However, atopic dermatitis remained uncontrolled. Six years later, dupilumab was initiated to manage atopic dermatitis while continuing omalizumab. This dual biologic approach demonstrated significant improvement in atopic dermatitis, prompting the discontinuation of omalizumab. However, omalizumab cessation coincided with asthma progression, necessitating the initiation of regular oral corticosteroid therapy. Therefore, omalizumab was reintroduced with continuing dupilumab. This combined regimen showed successful asthma management, making his current maintenance of omalizumab and dupilumab (Figure 2). The use of dual biologics demonstrated no adverse effect associations.

## DISCUSSION

Herein, we describe two cases of severe asthma with atopic dermatitis that were successfully managed through the initiation of dual biologic therapies. Dual biologics, particularly incorporating dupilumab, may be a good candidate for patients with both severe asthma and atopic dermatitis. Notably, this is the first case of severe asthma receiving dual biologics including tezepelumab and furthermore bronchial thermoplasty. In addition, we firstly present a comprehensive literature review on dual biologics to date, making a significant contribution to the existing knowledge base.

The important point of our investigation includes the safe and efficacious use of these dual biologic regimens. Dupilumab targets IL‐4/IL‐13 and improves both severe asthma and atopic dermatitis.^4^, ^6^ However, patients with atopic dermatitis tended to suffer from conjunctivitis after dupilumab administration.^17^ The use of mepolizumab in addition to dupilumab, which targets both IL‐4/13 and IL‐5 pathways, may improve atopic dermatitis and conjunctivitis, all while managing severe asthma in case 1. Tezepelumab, which targets TSLP, appears as a potential therapeutic option for severe asthma, irrespective of type 2 markers.^5^ Targeting both IL‐4/13 and TSLP pathways could have a synergetic effect; however, the effect may be similar to other dual biologics. The use of omalizumab in addition to dupilumab, which targets both IL‐4/13 and IgE pathways, may improve both atopic dermatitis and severe asthma in case 2. Dupilumab, in fact, reduces total IgE levels and impacts the IgE pathway.^4^ On the other hand, omalizumab plays a different role in the pathophysiology of asthma. It increases circulating regulatory T cells, reduces circulating free IgE levels and decreases the expression of type 1 Fcε receptors in dendritic cells, mast cells, basophils, and eosinophils. These mechanisms may account for the additive benefit observed when omalizumab was combined with dupilumab in case 2.^18^ These two cases demonstrated the advantages of dual biologics in patients who remain unmanaged by single biologic treatments, especially when complicated by comorbid conditions with evidence of other biologics. Additionally, these two cases indicated the benefit of concurrently targeting IL‐4/13 and other pivotal pathways such as IL‐5, TSLP, or IgE.

In case 1, bronchial thermoplasty was introduced after the initiation of dual biologics, resulting in improved asthma control. Bronchial thermoplasty has demonstrated a sustained reduction in severe asthma exacerbations over a period of 10 years.^19^ This long‐term effect may hold promise even for patients receiving dual biologic therapy. To our knowledge, this is the first case in which bronchial thermoplasty was combined with dual biologics. Consequently, it is imperative to closely monitor the safety as well as efficacy of incorporation of bronchial thermoplasty in the future.

To date, 38 cases (excluding conference papers) used dual biologics for severe asthma, as shown in Table 1.^9^, ^10^, ^11^, ^12^, ^13^, ^14^, ^15^, ^16^ These cases were aged 49 ± 16 years, consisting of approximately one‐third male. The effective rate of dual biologics for asthma was 21/25 (84%) while acknowledging potential publication bias. Comorbidities included only asthma in 15 (39%), chronic rhinosinusitis in 9 (24%), atopic dermatitis in 6 (16%), eosinophilic granulomatosis with polyangiitis in 5 (13%), chronic spontaneous urticaria in 4 (11%), allergic rhinitis in 4 (11%), and allergic bronchopulmonary aspergillosis in 3 (8%) patients. The most used combination was omalizumab and mepolizumab (42%). Dual biologics were administered due to asthma (58%), comorbidities (34%), or both (8%). Dual biologics are avoided because of concerns of serious infections in rheumatoid arthritis or inflammatory bowel diseases in the context of adverse effects.^20^, ^21^ However, our findings emphasized the safety of dual biologic administration for asthma management, in all the cases, including ours, that experience no serious adverse effects such as anaphylaxis, immune dysfunctions, or infections. Therefore, dual biologics may be considered for promising therapeutic option when patients have multiple potential therapeutic targets and a situation where a single biologic is inadequate. However, this field warrants further prospective evidence. Clinical trials for dual biologics should be performed for patients with severe asthma in whom a single biologic is insufficient for management, safety follow‐up should be included in registries, and a longer follow‐up should be performed. As the strategy of the use of dual biologics, we should initially attempt to switch to another biologic, and dual biologics are considered when each biologic responds partially but insufficiently to control the situation.

**TABLE 1:** Previous reports of dual biologics for asthma.

Case number	Authors	Age (y), sex	First biologics	Rotation trial	Added biologics	Reason for dual biologics	Allergic or eosinophilic comorbidities	Outcome	Adverse effect
Cases with observed effectiveness									
1	Altman et al.^9^	58, F	Omalizumab	No	Mepolizumab	Frequent asthma exacerbation	ABPA	Improved asthma	None
2	Dedaj et al.^10^	55, F	Omalizumab	No	Mepolizumab	Frequent asthma exacerbation	None	Improved asthma	None
3	Ortega et al.^11^	61, F	Omalizumab	No	Dupilumab	Remained refractory AD	AR, CRS, ABPM, AD	Improved AD	None
4	Ortega et al.^11^	60, F	Omalizumab	No	Mepolizumab → Benralizumab	Frequent asthma exacerbation	AR, CRS, CSU	Improved asthma	None
5	Ortega et al.^11^	43, M	Omalizumab	No	Mepolizumab → Benralizumab → Duplilumab	Frequent asthma exacerbation	CRS, ABPA, AC	Improved asthma	None
6	Domingo et al.^12^	55, F	Mepolizumab	Yes	Omalizumab	Appearance of clinical signs of allergy	AR	Improved allergy symptom	None
7	Fox et al.^13^	12, F	Omalizumab	No	Mepolizumab	Frequent asthma exacerbation	AR	Improved asthma	Mild headache
8	Briegel et al.^14^	24, F	Benralizumab	Yes	Dupilumab	Recurrence of nasal polyposis	EGPA	Improved nasal polyposis symptom	None
9	Lommatzsch et al.^16^	70, F	Benralizumab	Yes	Dupilumab	AD treatment	AD	Improved asthma	None
10	Lommatzsch et al.^16^	32, F	Benralizumab	Yes	Dupilumab	CRS treatment	CRS	Improved asthma	None
11	Lommatzsch et al.^16^	49, M	Dupilumab	Yes	Mepolizumab	Asthma treatment	CRS	Improved asthma	None
12	Lommatzsch et al.^16^	52, M	Benralizumab	Yes	Omalizumab	CSU treatment	CSU	Improved asthma	None
13	Lommatzsch et al.^16^	26, F	Benralizumab	Yes	Dupilumab	CRS treatment	EGPA, CRS	Improved asthma	None
14	Lommatzsch et al.^16^	83, F	Omalizumab	Yes	Mepolizumab	EGPA treatment	EGPA	Improved asthma	None
15	Lommatzsch et al.^16^	52, F	Mepolizumab	Yes	Dupilumab	CRS treatment	CRS	Improved asthma	None
16	Lommatzsch et al.^16^	39, M	Benralizumab	Yes	Dupilumab	CRS treatment	CRS	Improved asthma	None
17	Lommatzsch et al.^16^	44, M	Mepolizumab	Yes	Dupilumab	AD treatment	AD	Improved asthma	None
18	Lommatzsch et al.^16^	48, M	Omalizumab	Yes	Mepolizumab	Asthma treatment	None	Improved asthma	None
19	Lommatzsch et al.^16^	56, F	Omalizumab	Yes	Dupilumab	Asthma treatment	None	Improved asthma	None
20	Our case 1	52, M	Dupilumab	Yes	Mepolizumab → Benralizumab → Tezepelumab	Poor asthma control and deterioration of AD	CSU, AD	Improved AD and slightly improved asthma	None
21	Our case 2	41, M	Dupilumab	No	Omalizumab	Frequent asthma exacerbation and deterioration of AD	AD	Improved asthma and AD	None
Cases with no observed effectiveness									
22	Lommatzsch et al.^16^	54, M	Mepolizumab, Benralizumab	Yes	Omalizumab	Asthma treatment	None	Unchanged asthma	None
23	Lommatzsch et al.^16^	41, F	Omalizumab	Yes	Dupilumab	Asthma and AD treatment	AD	Unchanged asthma	None
24	Lommatzsch et al.^16^	55, M	Benralizumab	Yes	Dupilumab	Asthma treatment	EGPA	Unchanged asthma	None
25	Lommatzsch et al.^16^	25, F	Omalizumab	Yes	Mepolizumab, Reslizumab	Asthma treatment	None	Unchanged asthma	None
Cases of uncertain effectiveness									
26	Pitlick et al.^15^	53, F	Omalizumab	N.R.	Benralizumab	Asthma treatment	None	N.R.	None
27	Pitlick et al.^15^	75, F	Omalizumab	N.R.	Benralizumab	Asthma treatment	None	N.R.	None
28	Pitlick et al.^15^	45, F	Omalizumab	N.R.	Benralizumab	Asthma treatment	None	N.R.	None
29	Pitlick et al.^15^	27, F	Omalizumab	N.R.	Benralizumab	Asthma treatment	None	N.R.	None
30	Pitlick et al.^15^	40, M	Mepolizumab	N.R.	Dupilumab	CRS treatment	CRS	N.R.	None
31	Pitlick et al.^15^	52, F	Omalizumab	N.R.	Mepolizumab	EGPA treatment	EGPA	N.R.	None
32	Pitlick et al.^15^	63, F	Omalizumab	N.R.	Mepolizumab	Asthma treatment	None	N.R.	None
33	Pitlick et al.^15^	12, F	Omalizumab	N.R.	Mepolizumab	Asthma treatment	None	N.R.	None
34	Pitlick et al.^15^	73, F	Omalizumab	N.R.	Mepolizumab	Asthma treatment	None	N.R.	None
35	Pitlick et al.^15^	47, F	Omalizumab	N.R.	Mepolizumab	Asthma treatment	CSU	N.R.	None
36	Pitlick et al.^15^	71, F	Omalizumab	N.R.	Mepolizumab	Asthma treatment	None	N.R.	None
37	Pitlick et al.^15^	63, M	Omalizumab	N.R.	Mepolizumab	Asthma treatment	None	N.R.	None
38	Pitlick et al.^15^	51, M	Omalizumab	N.R.	Mepolizumab	Asthma treatment	None	N.R.	None

Abbreviations: ABPM, allergic bronchopulmonary mucosis; AC, allergic conjunctivitis; AD, atopic dermatitis; AR, allergic rhinitis; CRS, chronic rhinosinusitis; CSU, chronic spontaneous urticaria; EGPA, eosinophilic granulomatosis with polyangiitis; EOM, eosinophilic otitis media; N.R., not reported.

Cost should be also considered. Adding another biologic increases costs; however, this investment may be offset by a reduction in repeated emergency department visits or hospitalizations and productivity improvement.^22^ Therefore, dual biologics would be acceptable in terms of costs when careful selection is performed.

In conclusion, dual biologics, with dupilumab and other biologics, including tezepelumab, may be an effective therapeutic strategy for patients with both severe asthma and atopic dermatitis. Future prospective study is required to investigate the efficacy and safety of the combination of biologics because only case reports or case series have been reported.

## AUTHOR CONTRIBUTIONS

Takeshi Matsumoto drafted the manuscript. All authors interpreted the data, critically revised the manuscript for intellectual content, and approved the final version of manuscript.

## CONFLICT OF INTEREST STATEMENT

None declared.

## ETHICS STATEMENT

Written informed consents for publication have been obtained.

## Data Availability

Data available on request from the authors.
